# Supplementary material for: Bridging the gap between consciousness and matter: recurrent out-of-body projection of visual awareness revealed by the law of non-identity
Source: Integr Psychol Behav Sci. 2023 May 24;58(1):178–203. doi: 10.1007/s12124-023-09775-y (PMC10904448; doi:10.1007/s12124-023-09775-y)
Supplement: Supplementary file 1 — (PDF 474 kb) [file 12124_2023_9775_MOESM1_ESM.pdf]

Supplementary Information for

Bridging the Gap Between Consciousness and Matter: Recurrent Out-of-Body Projection of Visual Awareness Revealed by the Law of Non-Identity

Jinsong Meng*

*Corresponding author. E-mail: mengjinsong@uestc.edu.cn

This PDF file includes:

Supplementary Materials and Methods

Supplementary Text

Supplementary Figures 1 to 4

Supplementary Table 1

1 Supplementary Materials and Methods

This section includes detailed descriptions of two visual psychophysical experiments of perceiving night-shot still life under the pre-induced accommodation condition: Experiment 1, visual perception of a night-shot metal ball, and Experiment 2, visual perception of a night-shot randomly selected drawing in compliance with a single-blind procedure.

1.1 Participants

Eighteen volunteers participated in the experiments (14 males and four females, aged between 18 and 49 years, not colorblind), six of whom had normal vision, whereas the others had corrected-to-normal vision and had no known neurological or visual disorders. They were unaware of the specific aim of the study. The studies involving human participants were reviewed and approved by the Ethics Committee of the University of Electronic Science and Technology of China. The participants provided their written informed consent to participate in this study prior to inclusion in the experiment. The participant in Supplementary figures 3 and 4 gave written informed consent for publication of his photographs.

1.2 Objects to be observed

The objects to be observed included an orange metal ball and nine drawings (fig. 1). The orange ball had a diameter of 12 cm, and the drawings with black margins (L × W, 12 × 12 cm) were classified as circle, square, and triangle shapes. Each shape was available in red, green, and cyan. Each drawing was marked on the back with a unique number from 1 to 9.

1.3 Test equipment

The test equipment comprised a camera, an off-camera flash (Flash model DF-800II; Sidande Inc., Shenzhen, China), a pair of master-slave wireless flash triggers (Model WFC-02; Sidande Inc.,

Shenzhen, China) used to synchronize the actions between the camera and the off-camera flash, a specially-designed visual pre-induced accommodation circuit (VPAC) that comprised a lumen measurement circuit (LMC) and a crosshair display circuit (CDC) (fig. 2a and 2b), and a digital oscilloscope.

The LMC can measure the luminous intensity received at the center of the observed object using a light sensor (Model SFH 5711-2/3; OSRAM, Germany) with a comparable spectral sensitivity to human eyes, whereas the CDC can present a red crosshair, with a diameter of 2 cm, through eight light-emitting diodes (LEDs) after pressing the set button of the VPAC and can be automatically closed after the flash onset (fig. 2c). The digital oscilloscope, when connected with the two test points (TP) of VPAC, can sample and display the luminous intensity via the LMC. The surroundings of the crosshair of the CDC were obscured by a light barrier to prevent their light from shining on objects that were to be observed, thereby ensuring that any object that was to be observed remained unknown to the participant prior to the experiment.

Before the experiment, the experimenter linked the slave trigger to the flash, pointed the flash towards the object to be observed, and linked the two TP outputs (that is, TP1 and TP2) of VPAC to the digital oscilloscope.

1.4 Experiment 1: Visual perception of night-shot metal ball

First, the experimenter adhered the VPAC to the center of the ball that was fixed on the light-absorbing backdrop. The VPAC and ball were shielded by a black curtain, thereby obscuring the vision of the participant. Subsequently, one participant was seated 3 m away from the ball (fig. 3a). Thereafter, the lamp in the room was turned off and the curtain was withdrawn; the experimenter pressed the set button of the VPAC to display the crosshair and asked the participant to concentrate on the red crosshair for 5s (fig. 3b). The experimenter then pressed the button of the master trigger and the flash shined on the

ball (fig. 3c). The participant was instructed to report the position, shape and color of what he/she saw.

Repeated tests (n=18) showed that under the pre-induced accommodation condition, each participant correctly reported the position, shape and color of the metal ball after a single-pulse diffuse reflection stimulus lasting for 500 μs (Table S1).

1.5 Experiment 2: Visual perception of night-shot randomly selected drawing

In this experiment, each test was conducted in compliance with a single-blind procedure—a drawing was randomly selected from nine drawings, which was unknown to the participant in the dark before flash onset. For this purpose, 18 random numbers in uniform distribution (discrete) were generated in advance (none of the numbers exceeded 9 and any two adjacent numbers were different). The experiments were conducted as follows:

First, according to the first unused random number in the list, the experimenter sought out the drawing marked with the same number and fixed it on the light-absorbing backdrop. The experimenter adhered the VPAC to the center of the drawing, both of which were shielded by a curtain, thereby obscuring the vision of the participant. Subsequently, one participant was seated 3 m away from the drawing (fig. 4a). Thereafter, the lamp was turned off and the curtain was withdrawn; the experimenter pressed the set button of the VPAC and asked the participant to concentrate on the red crosshair for 5 s (fig. 4b). The experimenter then pressed the button of the master trigger and the flash shined on the drawing once (fig. 4c). The participant was instructed to report the position, shape and color of what he/she observed. Thus, a test task for one participant recognizing a randomly selected drawing was completed, and the random number was marked as "used."

Repeated tests (n=18) showed that under the pre-induced accommodation condition, each participant correctly reported the position, shape and color of a randomly selected drawing after a single-pulse diffuse reflection stimulus lasting for 500 μs (Table S1).

2 Supplementary Text

Although the home camera flash working at full power is safe for healthy participants, the experimenter applied a diffuser in front of the flash to soften the flash light and adjusted the output power of the flash to a lower level (here the output power was set to 1/64 of the full power). Furthermore, a dark blue filter was applied to cover the crosshair of the VPAC to attenuate the luminous intensity of the crosshair. These measures ensured that the participants were visually comfortable, and therefore, the potential negative impacts on participants in this study were even less than those of usual night photography.

3 Supplementary Figures

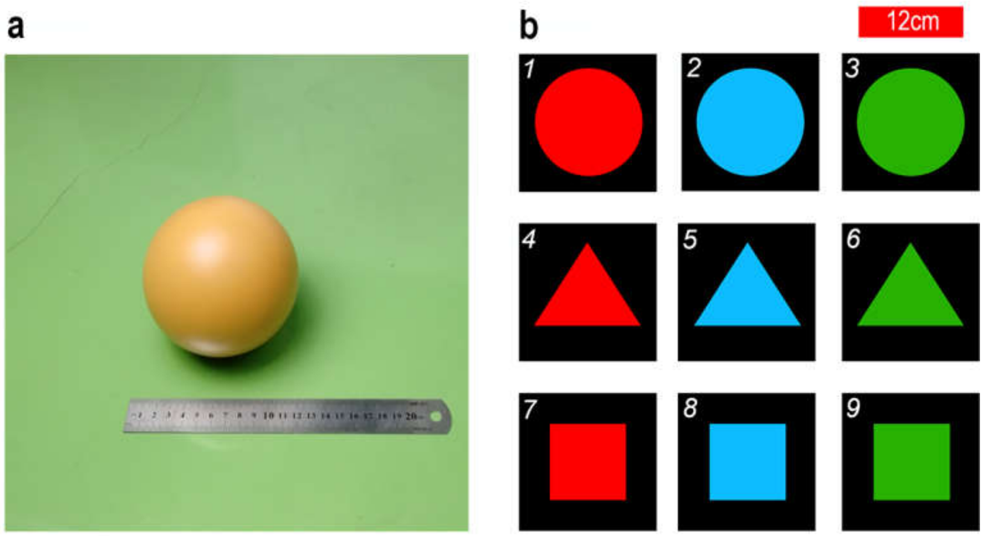

Fig. 1 Two types of objects to be observed. **a**, An orange metal ball with a diameter of 12 cm. **b**, Nine drawings of different shapes in different colors with black margins, the backs of which were marked with a unique number from 1 to 9.

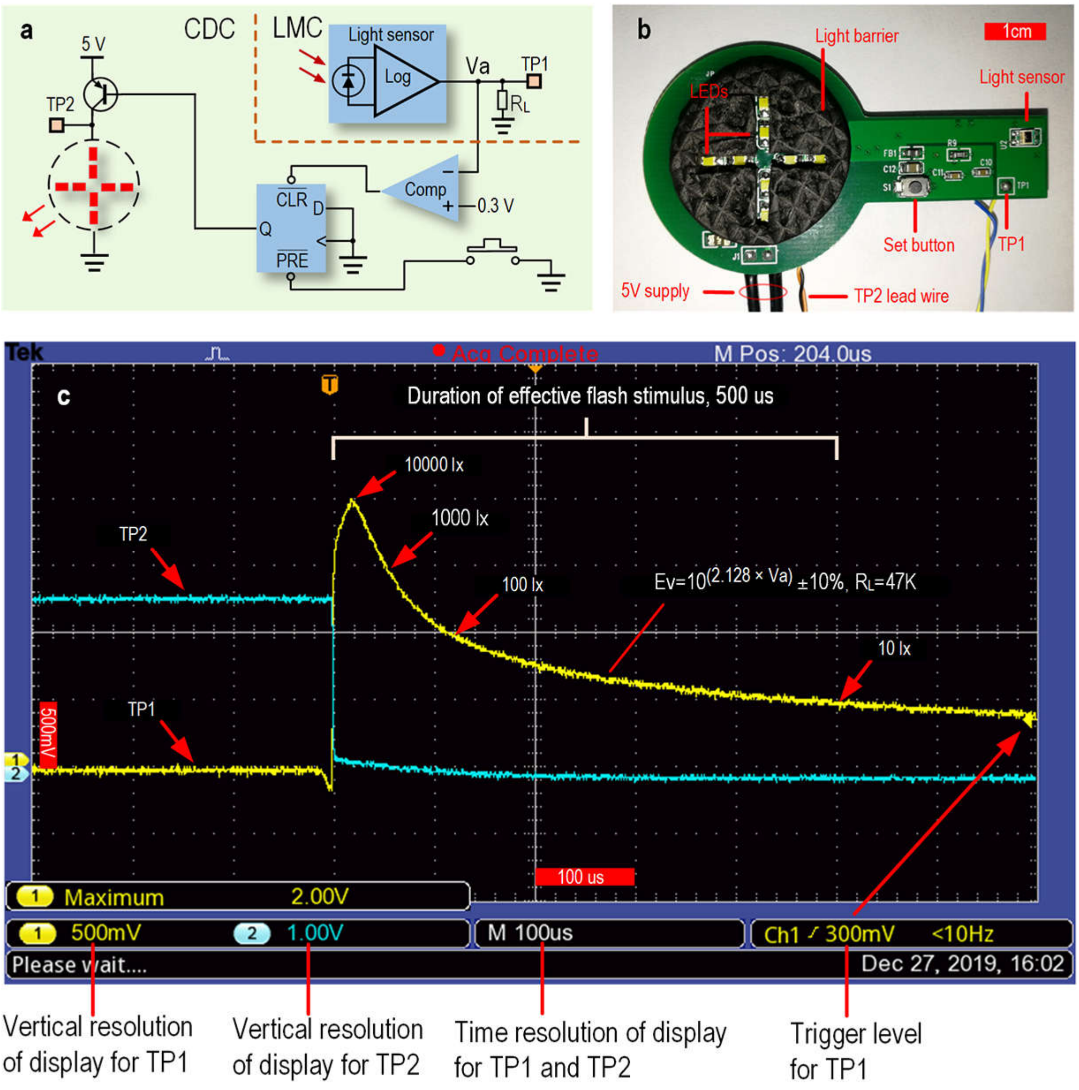

Fig. 2 Visual pre-induced accommodation circuit (VPAC) and its response to the flash. **a**, Schematic frame of the VPAC. The module comprises two simple circuits: crosshair display circuit (CDC) and lumen measurement circuit (LMC). In the dark, on clicking the set button, CDC can present a red crosshair until the LMC receives a single-pulse flash stimulus. When Va, the output voltage of the lumen sensor, exceeds the threshold of 0.3 V, the comparator can output a low level through a flip-flop to turn off the power switch of the light-emitting diodes (LEDs). **b**, Physical appearance of the VPAC. The light barrier, a trimmed, round, and self-adhesive furniture pad, with a height of 2 mm. A dark blue filter was used to cover the barrier, for attenuating the light of the LEDs (not shown). **c**, Response of the VPAC to a single-pulse flash. The duration of the flash was approximately 500 μs at 1/64 full power of the flash, whereas the red crosshair went out immediately after the flash onset.

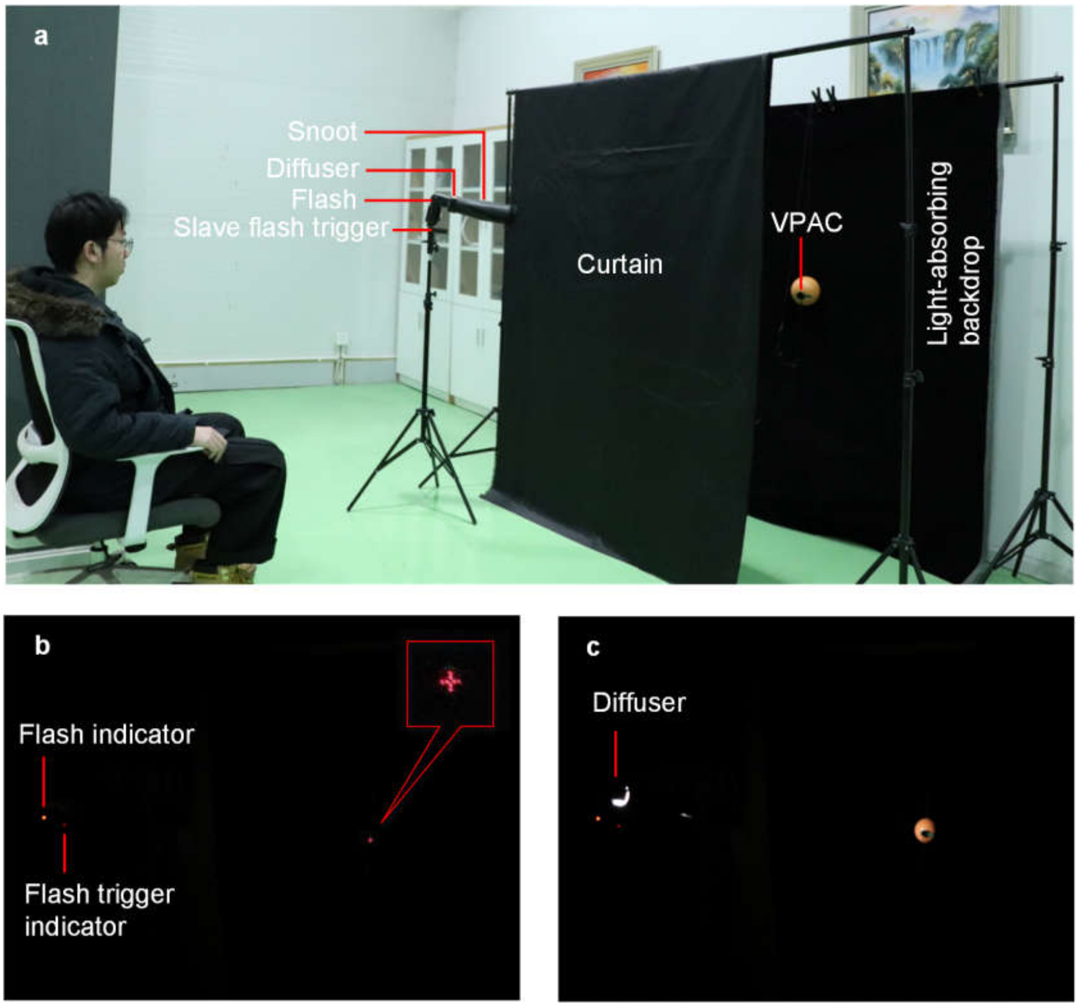

Fig. 3 Experimental procedure for recognizing the metal ball. **a**, Layout of the participant and equipment. The participant was seated 3 m away from the ball to be observed. **b**, After the curtain was withdrawn in the dark, the participant fixated on the red crosshair for 5s. **c**, After the experimenter pressed the button of the master trigger, the flash shined on the ball once.

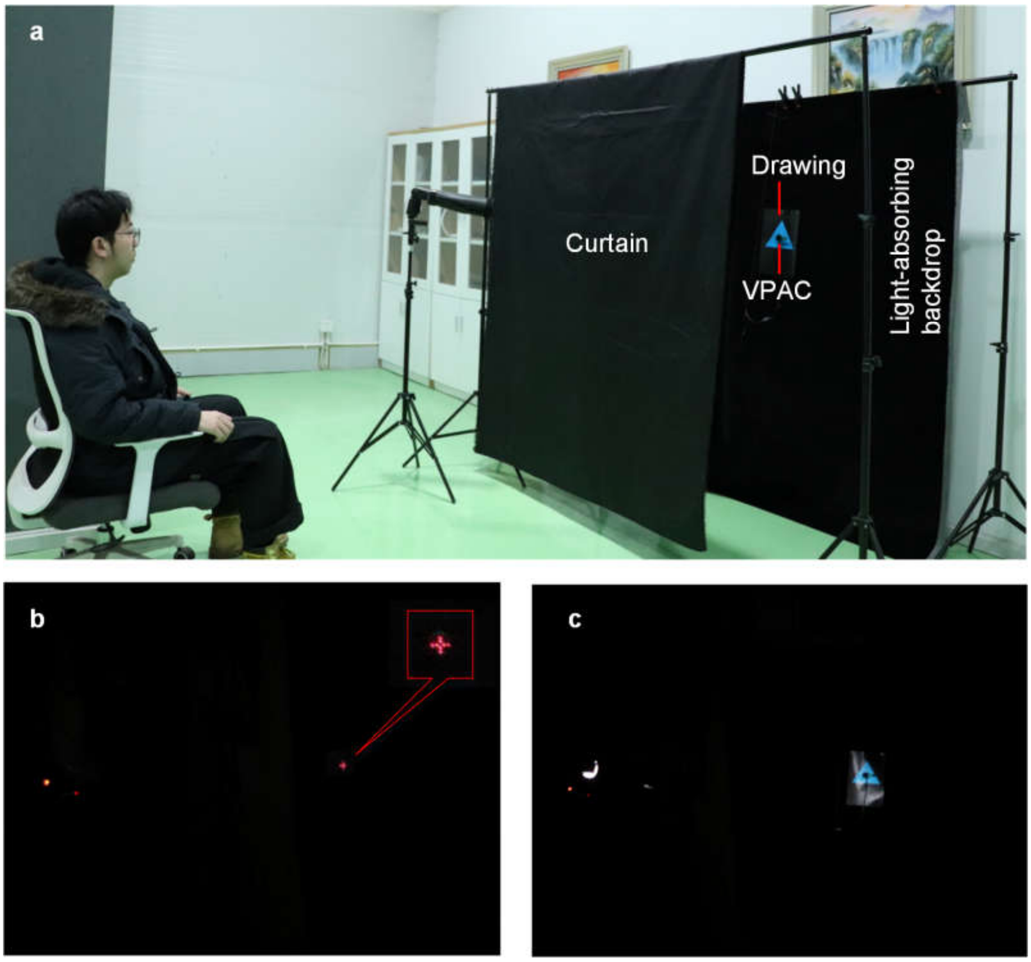

Fig. 4 Experimental procedure for recognizing a randomly selected drawing in compliance with a single-blind procedure. **a**, Layout of the participant and equipment. The participant was seated 3 m away from the drawing to be observed. **b**, After the curtain was withdrawn in the dark, the participant's gaze fixated on the red crosshair for 5 seconds. **c**, After the experimenter pressed the button of the master trigger, the flash shined on the drawing once. (**b** & **c,** the photos were taken behind the participants)

4 Supplementary Table

Table S1. Experimental result of participants recognizing the night-shot still life

	Observed object	Number of participants	Stimulation intensity	Stimulation duration	Recognition rate
Experiment 1	Metal ball	18	1/64 full power	500 µs	100%
Experiment 2[a]	Drawing	18	1/64 full power	500 µs	100%

[a]Each test in experiment 2 was conducted in compliance with a single-blind procedure—a drawing was randomly selected from nine drawings which was unknown to the participant in the dark before flash onset.
